# Clinical Outcomes following Large Vessel Coronary Artery Perforation Treated with Covered Stent Implantation: Comparison between Polytetrafluoroethylene- and Polyurethane-Covered Stents (CRACK-II Registry)

**DOI:** 10.3390/jcm10225441

**Published:** 2021-11-21

**Authors:** Jerzy Bartuś, Rafał Januszek, Damian Hudziak, Michalina Kołodziejczak, Łukasz Kuźma, Mateusz Tajstra, Tomasz Figatowski, Tomasz Pawłowski, Monika Gruz-Kwapisz, Malwina Smolarek-Nicpoń, Agnieszka Skoczyńska, Brunon Tomasiewicz, Adrian Włodarczak, Jan Kulczycki, Krzysztof Plens, Miłosz Jaguszewski, Sławomir Dobrzycki, Andrzej Ochała, Mariusz Gąsior, Krzysztof Reczuch, Stanisław Bartuś, Wojciech Wojakowski, Wojciech Wańha

**Affiliations:** 1 Second Department of Cardiology, Jagiellonian University Medical College, 31-501 Kraków, Poland; jerzy.bartus@gmail.com (J.B.); stanislaw.bartus@uj.edu.pl (S.B.); 2 Department of Clinical Rehabilitation, University of Physical Education, 31-571 Kraków, Poland; 3 Department of Cardiology and Cardiovascular Interventions, University Hospital, 30-688 Kraków, Poland; 4 Department of Cardiac Surgery, Medical University of Silesia, 40-635 Katowice, Poland; damhud@gmail.com; 5 Department of Anaesthesiology and Intensive Care, Ludwik Rydygier Collegium Medicum, Nicolaus Copernicus University, Antoni Jurasz University Hospital No. 1, 85-094 Bydgoszcz, Poland; kolodziejczak.michalina@gmail.com; 6 Department of Invasive Cardiology, Medical University of Białystok, 15-276 Białystok, Poland; kuzma.lukasz@gmail.com (Ł.K.); s.dobrzycki6219@gmail.com (S.D.); 7 Third Department of Cardiology, Medical University of Katowice, 41-800 Zabrze, Poland; mateusztajstra@wp.pl (M.T.); m.gasior@op.pl (M.G.); 8 First Department of Cardiology, Medical University of Gdansk, 80-210 Gdańsk, Poland; figatowski@gumed.edu.pl (T.F.); jamilosz@gmail.com (M.J.); 9 Department of Cardiology and Structural Heart Diseases, Medical University of Silesia, 40-635 Katowice, Poland; tomaszzpawlowski@gmail.com (T.P.); gruzmonika1@gmail.com (M.G.-K.); malwina.smolarek@gmail.com (M.S.-N.); askoczynska@wp.pl (A.S.); aochala1@gmail.com (A.O.); wojtek.wojakowski@gmail.com (W.W.); wojciech.wanha@gmail.com (W.W.); 10 Centre for Heart Disease, University Hospital Wrocław Department of Heart Disease, Wrocław Medical University, 50-556 Wrocław, Poland; b.a.tomasiewicz@gmail.com (B.T.); reczuch@poczta.wp.pl (K.R.); 11 Department of Cardiology, Miedziowe Centrum Zdrowia, 59-300 Lubin, Poland; wlodarczak.adrian@gmail.com (A.W.); jan.jakub.kulczycki@gmail.com (J.K.); 12 Kraków Cardiovascular Research Institute, 30-055 Kraków, Poland; plens_krzysztof@o2.pl

**Keywords:** coronary artery perforation, clinical outcomes, covered stents, PTFE and polyurethane stent comparison

## Abstract

Data on the clinical outcomes comparing synthetic fluorocarbon polymer polytetrafluoroethylene- (PTFE, GraftMaster) and polyurethane- (Papyrus) covered stents (CSs) to seal coronary artery perforations (CAPs) are limited. We aimed to evaluate 30-day and 1-year clinical outcomes after PCI complicated by CAP and treated with CS. We assessed 106 consecutive patients with successful CAP sealing (122 CSs): GraftMaster (51 patients, 57 CSs) or Papyrus CS (55 patients, 65 CSs). The primary endpoint was the occurrence of major adverse cardiac events (MACE), defined as the composite of cardiac death, target lesion revascularisation (TLR), and myocardial infarction (MI). The mean age of subjects was 69 ± 9.6 years (53.8% males). No significant differences were identified between the GraftMaster and Papyrus groups at the 30-day follow-up for MACE, cardiac death, MI and stent thrombosis (ST), while significantly lower rate of TLR and TVR (*p* = 0.02) were confirmed in the Papyrus group. At one year, differences remained similar between stents for MACE, a trend towards a lower rate of TLR (*p* = 0.07), MI (*p* = 0.08), and ST (*p* = 0.08), and higher for cardiac death (*p* = 0.07) was observed in the Papyrus group. This real-life registry of CAP illustrated that the use of Papyrus CS is associated with lower rates of TLR and TVR at 30-day follow-up in comparison to the GraftMaster CSs and no significant differences between both assessed CS at one year of follow-up.

## 1. Introduction

Coronary artery perforation (CAP) is a rare periprocedural complication, which occurs in 0.17–0.43% of patients during percutaneous coronary intervention (PCI) [1,2]; the incidence increases to 4.1–4.8% in procedures performed on complex lesions [3,4]. The Ellis classification remains a recommended tool that stratifies CAP severity and guides through its management [5]. Mild perforations are usually sufficiently treated with prolonged balloon inflation or additional stent implantation. Advanced techniques that include the use of coagulation reversal agents, blood component transfusion, urgent cardiac surgery, or covered stent (CS) implantation are applied in case of life-threatening CAPs of the large vessels with haemodynamic instability and resistance against standard treatment [6]. In previous research, it was shown that in CSs, satisfactory safety is exhibited as well as efficacy profile for the management of CAPs during PCI [7]. The construction of CS consists of a metallic stent platform covered with a synthetic (expanded polytetrafluoroethylene (GraftMaster) or electrospun polyurethane (Papyrus)) or biological membrane (pericardium) that seals the blood extravasation [7]. The differences in the type of stent cover and structure (sandwich or out-/inlayer cover) impact the endothelialisation process that prolongs stent thrombogenicity potency, similarly to other implantable devices [7,8]. This biocompatibility translates into a significantly higher risk of adverse events with CSs as compared to drug-eluting stents (DES), but not to bare metal stents (BMS) [9,10]. In previous studies, there were no broad reports on the effect of variance within CS structure type. To address this evidence gap, we conducted a study focusing on one-year results and predictors of clinical outcomes of CAP patients treated with GraftMaster and Papyrus implantation.

## 2. Methods

A detailed description of the multicentre, observational CRACK Registry (NCT04630314) design has been previously presented [11]. The dataset included consecutive patients with iatrogenic, peri-PCI CAP, treated with CS implantation between January 2009 and October 2019, at 8 high-volume PCI centres. Outcome data were obtained from the central database of the National Health Fund Service of the Ministry of Health, asserting follow-up completion for all patients. If re-PCI or coronary artery bypass surgery occurred during the follow-up period, data on target vessel revascularisation (TVR) and target lesion revascularisation (TLR) were collected. Data were anonymised at the level of each centre, merged into a single database, and statistically analysed. The study was approved by the appropriate local ethical committees. The patient’s data were protected according to the requirements of Polish law, General Data Protection Regulation (GDPR) and hospital Standard Operating Procedures. The study was conducted in accordance with the Declaration of Helsinki. 

### 2.1. Evaluated Covered Stents and Structural Insight

Two types of CS were evaluated: GraftMaster CS and Papyrus CS. The main technological differences between the two stents are graft material and stent design. The GraftMaster (Abbott Vascular, Santa Clara, CA, USA) CS is constructed using the sandwich technique, whereby a layer of expanded polytetrafluoroethylene (ePTFE) is placed between two stainless steel stents. The study stent was available in sizes 3–4 mm, 6–7 Fr guide catheter compatible. The PK Papyrus (Biotronik, Bülach, Switzerland) CS is a single layer 90-µm electrospun polyurethane-covered cobalt-chromium stent. The study stent was available in sizes 2.5–5 mm, 5–6 Fr guide catheter compatible.

### 2.2. Procedure

The PCI procedure with CS implantation and antithrombotic treatment was at the operator’s discretion, in accordance with the recommendations of the guidelines for clinical practice [12].

### 2.3. Angiography Analysis

Morphology of the stented lesion was defined according to the classification proposed by the ACC/AHA (American College of Cardiology/American Heart Association) [13]. Lesion length, percentage diameter stenosis, and the coronary flow (thrombolysis in myocardial infarction—TIMI classification) were assessed in all patients. Measurements were performed by two experienced interventional cardiologists based on cine coronary angiography at each site, on the angiograph available in catheterization laboratory, and interim quantitative coronary angiography dedicated to the particular angiograph. The three-staged Ellis classification was used to determine the degree of perforation based on angiographic manifestation [5].

### 2.4. Patient Follow-Up and Study Endpoints

The primary endpoint was the composite of major adverse cardiac events (MACE) defined as cardiac death, target lesion revascularisation (TLR) and myocardial infarction (MI) assessed at 30 days and 1 year from the index procedure. The secondary endpoints were stent thrombosis (ST) and the individual events of the primary composite endpoint. TLR was defined as any revascularisation within the treated lesion. ST was defined as acute (0–24 h post stent implantation), subacute (from 24 h to 30 days post stent implantation), or late (from 30 days to 1 year after stent implantation) [14].

### 2.5. Statistical Analysis

Categorical variables are presented as numbers and percentages. Continuous variables are expressed as mean ± standard deviation (SD). Normality was assessed by the Shapiro-Wilk test. Equality of variances was assessed using Levene’s test. Differences between groups were compared using the student’s or Welch’s *t*-test depending on the equality of variances for normally distributed variables. The Mann–Whitney U-test was used for non-normally distributed continuous variables. Ordinal variables were compared using the Cochran–Armitage test for trend or the Mann–Whitney U-test. Nominal variables were compared via Pearson’s chi-squared test or Fisher’s exact test if 20% of cells had an expected count of less than 5.

To analyse event-free survival in selected risk groups, Kaplan–Meier curves were drawn. The log-rank statistic was used to test differences in outcomes between the groups. Determinants of MACE, cardiac death, TLR, TVR, re-PCI, and myocardial infarction were determined by univariate and multivariate Cox regression models. The multivariate models were adjusted according to age, sex and BMI. Statistical analyses were performed with JMP^®^, Version 16.0.0 (SAS Institute INC., Cary, NC, USA).

## 3. Results

### 3.1. Baseline Clinical Characteristics

The registry included 106 patients with CAP, among which 51 (48.1%) received Graft Master CS and 55 (51.9%) Papyrus CS. There were no significant differences in age, gender or concomitant diseases between the GraftMaster and Papyrus groups, except for the atrial fibrillation, which occurred significantly more often in the Papyrus group (11.8% vs. 29.1%, *p* = 0.03), as well as greater borderline frequency of prior myocardial infarction in the GraftMaster group (43.1% vs. 25.5%, *p* = 0.05). Moreover, patients from the Papyrus group where qualified for PCI significantly more often due to non ST-segment elevation myocardial infarction (NSTEMI) (32.7% vs. 11.8%, *p* = 0.01) (Table 1).

### 3.2. Angiographic and Culprit Lesion Characteristics

Patients who experienced CAP within the right coronary artery during PCI significantly more often received Papyrus CS PCI when compared to the GraftMaster group (32.7% vs. 15.7%, *p* = 0.04). There were no differences in any of the remaining selected indices (Table 2).

### 3.3. Procedural Indices

Lesion predilatation was more frequently performed in the GraftMaster compared to Papyrus group (26.5% vs. 5.5%, *p* = 0.003). The mean maximum pressure of non-CS stent deployment was significantly greater in the Papyrus group (*p* = 0.01), as well as balloon predilatation mean maximum pressure (*p* = 0.003). For patients receiving Papyrus CS, the mean stent graft length was greater compared to the GraftMaster group (*p* = 0.004) (Table 2).

### 3.4. Coronary Artery Perforation and Periprocedural Complications

There were no significant differences in the class of Ellis type perforation between the GraftMaster and Papyrus groups for class 1 (31.4% vs. 30.9%, *p* = 0.96), 2 (19.6% vs. 29.1%, *p* = 0.26), and 3 (49% vs. 40%, *p* = 0.35). Administration of protamine sulphate was more frequent in the Papyrus group when compared to GraftMaster (30.9% vs. 2%, *p* < 0.001). Cardiac tamponade occurred in 44 patients (41.5%) at a significantly higher frequently than in the GraftMaster group when compared to the Papyrus group (52.9% vs. 30.9%, *p* = 0.02) (Table 3).

### 3.5. Clinical Endpoints and Follow-Up

Considering the number of patients during follow-up, fifteen patients died up to a year (cardiac death from day 0 to day 358), 18 were followed up to a year (less than day 365, day 21 to day 350). This can be seen in the KM charts, because on day 365, 73 patients are at risk.

No statistically significant differences were found between the GraftMaster and Papyrus groups in the occurrence of re-PCI, cardiac death, myocardial infarction or MACE during the 30-day follow-up. Papyrus CS implantation resulted in a significantly lower incidence of TVR and TLR, when compared to GraftMaster CS (Table 4).

**Figure 1:**
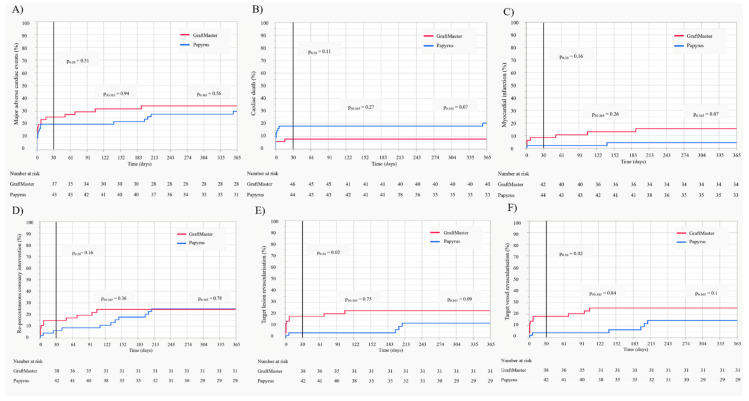
Kaplan–Meier estimates with the follow-up at 30 days and 1 year. (**A**) Major adverse cardiac events (MACE); (**B**) cardiac death; (**C**) myocardial infarction; (**D**) recurrent percutaneous coronary intervention (re-PCI); (**E**) target vessel revascularisation (TVR); (**F**) target lesion revascularisation (TLR).

At the 1-year follow-up, there were no statistically significant differences between GraftMaster and Papyrus CSs in the prevalence of MACE or re-PCI, with a trend towards lower rates of TLR, TVR, MI and ST in Papyrus CS. A trend towards a lower rate of cardiac death was observed in the group managed by GraftMaster stent implantation (Table 4). We observed a trend towards a lower overall ST rate in the Papyrus group compared to the GraftMaster group during the 1-year follow-up period (9.8% vs. 1.8%, *p* = 0.08; Table 4). Kaplan–Meier estimates for the selected clinical endpoints with the follow-up at 30-days and 1-year are presented in Figure 1A–E.

### 3.6. Predictors of Clinical Outcomes

**Figure 2:**
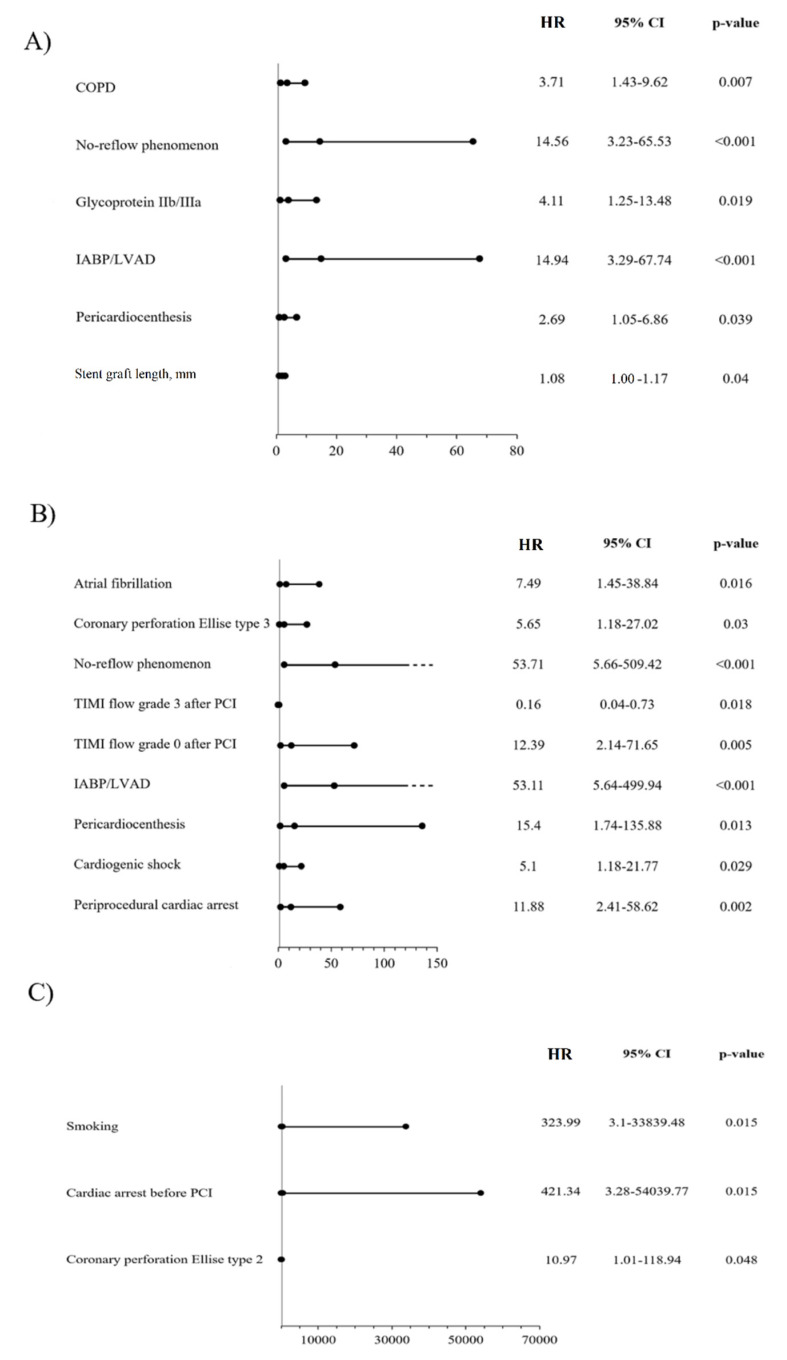
Predictors of selected clinical outcomes assessed by multivariable analysis-models adjusted for age, sex and BMI; (**A**) major adverse cardiac events (MACE); COPD, chronic obstructive pulmonary disease; IABP, intra-aortic balloon pump counter-pulsation; LVAD, left ventricle assist device. (**B**) Cardiac death; IABP, intra-aortic balloon pump counter-pulsation; LVAD, left ventricle assist device; PCI, percutaneous coronary intervention; TIMI, thrombolysis in myocardial infarction. (**C**) Myocardial infarction; PCI, percutaneous coronary intervention.

**Figure 3:**
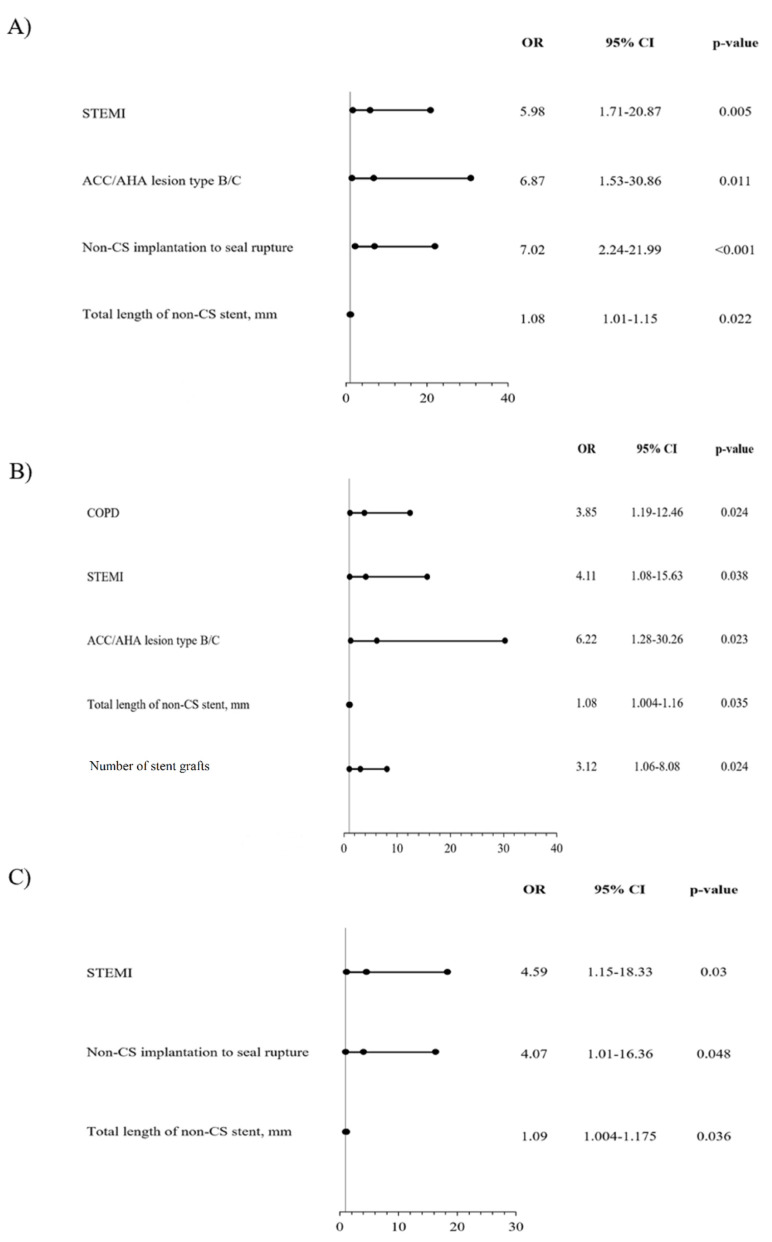
Predictors of selected clinical outcomes assessed by multivariable analysis-models adjusted for age, sex and BMI; (**A**) recurrent percutaneous coronary intervention (re-PCI); ACC/AHA, American College of Cardiology/American Heart Association; CS, covered stent; STEMI, ST-segment elevation myocardial infarction. (**B**) Target vessel revascularisation (TVR); ACC/AHA, American College of Cardiology/American Heart Association; CS, covered stent; COPD, chronic obstructive pulmonary disease, ST- segment elevation myocardial infarction. (**C**) Target lesion revascularisation (TLR); CS, covered stent; STEMI, ST- segment elevation myocardial infarction.

A number of significant predictors of 1-year MACE were confirmed by multivariable analysis (with adjustment for age, sex and BMI): chronic obstructive pulmonary disease, no-reflow phenomenon, treatment with GPIIb/IIIa inhibitor, periprocedural use of left ventricular assist device (IABP [intra-aortic balloon counter-pulsation], Maquet Cardiopulmonary AG, Rastatt, Germany; Impella, CP 5.0 L, Abiomed MA; extracorporeal circulation), pericardiocentesis and stent graft length (Figure 2A). Among significant predictors of cardiac death, the following were found: atrial fibrillation, CAP type 3, no-reflow phenomenon, TIMI flow grade 0 after PCI, periprocedural use of left ventricular assist device, pericardiocentesis, cardiogenic shock, and periprocedural cardiac arrest (Figure 2B). The occurrence of myocardial infarction during the follow-up period was significantly influenced by smoking, cardiac arrest prior to PCI and CAP type 3 (Figure 2C). Predictors of re-PCI included: ACC/AHA lesion type B/C, non-CS implantation to seal the rupture and total length of non-CS stent (Figure 3A). Among predictors of TVR, the following were confirmed: chronic obstructive pulmonary disease, ST-segment elevation myocardial infarction (STEMI), ACC/AHA lesion type B/C, total length of non-CS stent and stent graft number (Figure 3B). Predictors of TLR included STEMI, non-CS implantation to seal rupture, and total length of non-CS stent (Figure 3C).

## 4. Discussion

The main findings of the presented CRACK-II registry analysis are that: (1) Papyrus CS implantation resulted in a lower 30-day incidence of TVR and TLR when compared to GraftMaster CS; the early occurrence of re-PCI, cardiac death, MI and MACE remained indifferent between the compared groups; (2) at the 1-year follow-up, Papyrus and GraftMaster CSs implantation resulted in a similar prevalence of MACE and re-PCI, with a trend towards lower rate TLR, TVR, MI, and ST rates with the use of Papyrus, and a trend towards a lower rate of cardiac death with GraftMaster CS implantation.

The results of our analysis allowed demonstrating a difference in the prevalence of clinical events between the compared covered stents, both over short- and long-term observation periods. The covered stents did not differ in implantation technique, but in their technical design from which the difference in the prevalence of clinical events may have originated.

The PTFE-covered stent construction predisposes to increased neointimal hyperplasia and subsequent delayed endothelization at the proximal and distal edges, resulting in a magnified risk of in-stent restenosis [15,16,17,18]. In the previous reports, in-stent restenosis was a prevalent complication in patients treated with CS implantation, reaching 31.6% of interventional cases observed at a mean follow-up of 159 ± 49 days, with the edge in-stent restenosis constituting 29.8% out of those cases [15]. In our registry, the significant benefit of Papyrus CS over GraftMaster on TLR and TVR was present over a short-term observation and was no longer maintained during a long-term follow-up, whilst we observed only a trend for this difference. This interventional benefit did not translate into a reduced risk of recurrent PCI or MACE with Papyrus CS, showing that both stents were equally effective with the management of coronary artery perforations and asserted completion of the index procedure. Importantly, based on our previous data, the non-CS implantation to seal the rupture was found to be a predictor of increased re-PCI and TLR rates [11], while in previously published studies, it was suggested that among techniques used for the lower rate of ST and in-stent restenosis, non-CS stent (DES) implantation on the edge of CS could be implemented [19]. In the current study, localisation of non-CS was not aimed at covering the CS edge and it was carried out prior to CS implantation; therefore, it could not be found as deliberate CS stent protection against in-stent restenosis.

In previous studies, it has been demonstrated that equine pericardium-covered stent implantation resulted in a lower rate of ST over the course of treatment compared to PTFE and polyurethane-covered devices [9]. The stents used in the current registry were constructed with electrospun polyurethane-covered cobalt-chromium (Papyrus) and expanded polytetrafluoroethylene (ePTFE, GraftMaster). Literature data are not available for comparisons of treatment outcomes between Papyrus and GraftMaster stents. The current work is the first attempt at such a comparison. Harnek et al. evaluated treatment effects between implanted CSs, but the results did not allow an objective comparison of Papyrus and GraftMaster, as the author focuses mainly on the comparison with equine pericardium-covered stents [9]. Although the groups of patients were larger compared to the study presented by our team (199 GraftMaster and 74 Papyrus CS), the follow-up periods differed in length, which impaired proper interpretation of the results [9]. Nevertheless, from the presented graphs it can be concluded that the mortality rate was higher in the Papyrus group, and the ISR frequency was more prevalent in the GraftMaster group. Taking into account the difference in outcomes between the two assessed stents, the aetiology should be initially sought in the structure of the stent and effect on the endothelialisation rate [8]. While the prevalence of subacute ST in patients after GraftMaster implantation varied in previous studies between 5.7% at 5 months [15] and 3.6% at the 9-month follow-up [19], in our study, lower rates of subacute in-CS ST have been demonstrated. At the same time, the length of dual-antiplatelet therapy was longer in the GraftMaster group when compared to the Papyrus CS, which, considering the difference between event rates, underlines its sub-effective ST management. We explored a number of other factors that could have influenced the ST in the presented study. The frequency of urgent blood transfusion, known for prothrombotic properties, did not differ between CS groups. The use of protamine sulphate also had no influence on the prevalence of ST, as it was significantly more often administered in the Papyrus when compared to the GraftMaster population. The length of CS is another factor potentially influencing the frequency of ST, while the length of the Papyrus stent was significantly greater when compared to the GraftMaster CS, which could potentially increase the rate of ST in Papyrus group, but not in GraftMaster one.

In this study, valuable insight was also provided on the predictors of clinical events in the analysed population. Factors determining angiographic PCI success (no-reflow phenomenon and post-PCI TIMI flow grade) and indicators of patients’ poor periprocedural condition (use of left ventricle support devices, cardiogenic shock, cardiac arrest, or pericardiocentesis) were identified as predictors of cardiac death. The higher rate of cardiac death with Papyrus when compared to GraftMaster CS could result from clinical state of patients treated with CS at baseline. This reflects the significantly greater rate of NSTEMI patients and a trend towards a lower percentage of patients with stable angina in the Papyrus compared to the GraftMaster group. Although CAP 3 type and atrial fibrillation were found to be significant predictors of increased death rate in the current analysis, initially, both analysed groups differed statistically significantly only in the case of atrial fibrillation. The greater proportion of patients with atrial fibrillation at baseline in the Papyrus group may be associated with more frequent use of anticoagulation and a greater risk of periprocedural bleeding complications when compared to the GraftMaster one. Smoking and cardiac arrest before PCI were the two greatest pre-procedural predictors of myocardial infarction at the 1-year follow-up, closely followed by procedural type 2 CAP (according to Ellis classification). While assessing revascularisation outcomes (re-PCI, TVR and TLR), their greater prevalence was predicted by lesion type according to the ACC/AHA classification and stent graft length, non-CS implantation to seal rupture and total length of non-CS, as well as STEMI presentation at baseline.

A number of MACE predictors were identified via multivariable analysis, including occurrence of the no-reflow phenomenon. In some patients, this could be related to greater inflation pressures during stent deployment or post-dilatation, which appears after squeezing of embolic material, mostly during post-dilatation, resulting in distal embolization, and is called the “toothpaste effect” [20]. Moreover, the periprocedural use of glycoprotein IIb/IIIa receptor inhibitors might be related to the no-reflow phenomenon and the following increased rate of MACE components during the follow-up period. Among other factors, indicators of patients’ poorer clinical states were calculated, such as left ventricle support devices or pericardiocentesis. Among the well-known factors of poorer clinical outcomes after PCI with stent implantation, the following was found: stent graft length, a relationship that has been previously demonstrated in several publications [21].

The topic of the modernity of CSs should also be discussed. There is no doubt that Papyrus CS was constructed in a modern way and belong to the group of new CSs, approved for use by the Food and Drug Administration for commercial use in the United States of America in 2018 [22]. Whereas, the GraftMaster CS was designed much earlier, and its sandwich structure dates back much earlier, and was known as Jostent, primarily, while the currently available stents have been approved for commercial use in 2017 by the Food and Drug Administration [23]. Therefore, it could be also considered in comparison different nomenclature: the early double-layer sandwich design GraftMaster versus new generation single layer Papyrus CSs. However, this is not entirely true, as the study covers the period in which the availability of stents was equal and still is, and the selection of stents was random, because as a rule, one catheterisation laboratory is not supplied with both CSs. The factors that determined the comparable consumption and availability of CSs were certainly price, frequency of use, or local preferences and habits.

In conclusion, this real-life registry of CAP illustrated that the use of Papyrus CS is associated with lower rates of TLR and TVR at 30-day follow-up in comparison to the GraftMaster CSs and no significant differences between both assessed CS at one year of follow-up.

### Study Limitations

There are several limitations of this study. First, we had no data on intravascular imaging and, thus, the mechanism of recorded ST is unknown. Secondly, there was a lack of quantitative findings, such as reference vessel diameter or minimal lumen diameter, which would have allowed for stratified exploratory data analyses. There was also no central core laboratory or unified quantitative coronary angiography assessment by dedicated software. Moreover, we were not able to assess the extent of endothelialisation and its distribution in the target vessel after CS implantation at selected time points following the PCI. Due to the nature of CS implantation (urgent and salvage mode), a prospective analysis could not be performed. Moreover, among the limitations, are the small sample size, and probably the low number of events that could make it difficult to perform meaningful outcome analyses.

## Figures and Tables

**Table 1:** General characteristics and left ventricle ejection fraction.

Selected Indices	Total *n* = 106	GraftMaster *n* = 51	Papyrus *n* = 55	*p*-Value
Age, years	69.05 ± 9.6	68.7 ± 9.5	69.3 ± 9.8	0.74
Gender, males	57 (53.8)	26 (51)	31 (56.4)	0.57
BMI, kg/m^2^	26.1 ± 5.4	26.6 ± 5.8	25.7 ± 5	0.8
Chronic kidney failure	27 (25.5)	13 (25.5)	14 (25.4)	0.99
Diabetes mellitus	32 (30.2)	16 (31.4)	16 (29.1)	0.79
Arterial hypertension	81 (76.4)	39 (76.5)	42 (76.4)	0.98
Hyperlipidaemia	71 (67)	35 (68.6)	36 (65.5)	0.72
COPD	15 (14.3)	10 (20)	5 (9)	0.11
Atrial fibrillation	22 (20.7)	6 (11.8)	16 (29.1)	0.03
Smoking	35 (33)	14 (27.5)	21 (38.2)	0.24
Prior myocardial infarction	36 (34)	22 (43.1)	14 (25.5)	0.05
Prior PCI	38 (35.9)	22 (43.1)	16 (29.1)	0.13
Prior CABG	13 (12.3)	6 (11.8)	7 (12.7)	0.88
Neoplasm	7 (6.6)	5 (9.8)	2 (3.6)	0.25
Clinical presentation				
Chronic coronary syndrome	41 (38.7)	24 (47.1)	17 (30.9)	0.09
Unstable angina	18 (17)	8 (15.7)	10 (18.2)	0.73
NSTEMI	24 (22.6)	6 (11.8)	18 (32.7)	0.01
STEMI	23 (21.7)	13 (25.5)	10 (18.2)	0.36
LVEF	48.8 ± 12.4	48.9 ± 11.4	48.8 ± 13.4	0.89

Data are presented as mean ± standard deviation or count (percentage). CABG, coronary artery bypass grafting; COPD, chronic obstructive pulmonary disease; BMI, body mass index; LVEF, left ventricle ejection fraction; NSTEMI, non ST-segment elevation myocardial infarction, STEMI, ST-segment elevation myocardial infarction.

**Table 2:** Coronary angiography and culprit lesion characteristics.

Selected Indices	Total *n* = 106	GraftMaster *n* = 51	Papyrus *n* = 55	*p*-Value
Radial vascular access	70 (66)	31 (60.8)	39 (70.9)	0.27
Coronary angiography				
Single-vessel disease	44 (41.5)	20 (39.2)	24 (43.6)	0.64
Two-vessel disease	41 (38.7)	21 (41.2)	20 (36.4)	0.61
Three-vessel disease	19 (17.9)	9 (17.6)	10 (18.2)	0.94
Location of culprit lesion				
Left main coronary artery	5 (4.7)	1 (2)	4 (7.3)	0.36
LAD	54 (50.9)	30 (58.8)	24 (43.6)	0.12
Circumflex coronary artery	18 (17)	7 (13.7)	11 (20)	0.39
Right coronary artery	26 (24.5)	8 (15.7)	18 (32.7)	0.04
SvG	9 (8.5)	6 (11.8)	3 (5.5)	0.31
Type of stenosis				
De novo lesion	97 (91.5)	44 (86.3)	53 (96.4)	0.08
Thrombosis	1 (0.9)	1 (2)	0 (0)	0.48
In-stent restenosis	9 (8.5)	7 (13.7)	2 (3.6)	0.08
ACC/AHA lesion type				
- A	3 (2.8)	1 (2)	2 (3.6)	1
- B	36 (34.0)	18 (35.3)	18 (32.7)	0.78
- B/C	28 (26.4)	12 (23.5)	16 (29.1)	0.52
- C	39 (36.8)	20 (39.2)	19 (34.6)	0.62
Severe calcification	29 (27.4)	10 (19.6)	19 (34.6)	0.08
Degree of stenosis	88.2 ± 11.4	88.5 ± 10.7	87.9 ± 12	0.91
Tortuosity	12 (11.3)	4 (7.8)	8 (14.6)	0.28
Length of stenosis	27.3 ± 14.1	23.4 ± 9.1	30.1 ± 16.5	0.1
Length of stenosis ≥20 mm	55 (71.4)	22 (66.7)	33 (75)	0.42
Bifurcation	14 (13.2)	6 (11.8)	8 (14.6)	0.67
Chronic total occlusion	6 (5.7)	3 (5.9)	3 (5.5)	1
Type of PCI				
Drug-eluting stent	73 (70.2)	28 (57.1)	45 (81.8)	0.006
Bare-metal stent	10 (9.6)	7 (14.3)	3 (5.5)	0.18
Plain-old balloon angioplasty	16 (15.4)	13 (26.5)	3 (5.5)	0.003
Bioresorbable scaffold	5 (4.8)	1 (2)	4 (7.3)	0.37
Rotablation	8 (7.6)	3 (5.9)	5 (9.1)	0.72
Intravascular ultrasound	4 (3.9)	0 (0)	4 (7.3)	0.12
Number of non-CS				
0	20 (19.1)	13 (25.5)	7 (13)	0.11
1	48 (45.7)	22 (43.1)	26 (48.2)	
2	33 (31.4)	15 (29.4)	18 (33.3)	
3	3 (2.9)	1 (2)	2 (3.7)	
4	1 (1)	0 (0)	1 (1.9)	
Non-CS length, mm	26.7 ± 8.6	25 ± 8.2	28.02 ± 8.7	0.17
Non-CS diameter, mm	3.45 ± 0.8	3.37 ± 0.6	3.5 ± 0.9	0.58
Non-CS deployment max. pressure, atm.	14.8 ± 2.9	13.7 ± 2.6	15.5 ± 2.9	0.01
Balloon predilatation	89 (84.8)	41 (80.4)	48 (88.9)	0.23
Balloon predilatation max. pressure, atm	15.3 ± 4.6	13.6 ± 3.4	17.1 ± 5.1	0.003
Balloon postdilatation	36 (34.3)	21 (41.2)	15 (27.8)	0.15
Number of stent grafts				
1	92 (86.8)	45 (88.2)	47 (85.5)	0.23
2	12 (11.3)	6 (11)	6 (10.9)	
3	2 (1.9)	0 (0)	2 (3.6)	
Stent graft length, mm	18.9 ± 4.5	18.3 ± 4.6	19.4 ± 4.3	0.004
Stent graft diameter, mm	3.3 ± 0.5	3.2 ± 0.4	3.3 ± 0.54	0.87
Pressure, atm	15.4 ± 4.2	14.5 ± 3.1	16.1 ± 4.7	0.09
Inflation time, s	21.5 ± 25.9	20.5 ± 14.5	21.9 ± 29.4	0.84

Data are presented as mean ± standard deviation or count (percentage). ACC, American College of Cardiology; AHA, American Heart Association; CS, covered stent; LAD, left anterior descending coronary artery; PCI, percutaneous coronary intervention; SvG, saphenous vein graft.

**Table 3:** Characterisation of coronary artery perforation, periprocedural complications, and their treatment.

Selected Indices	Total *n* = 106	GraftMaster *n* = 51	Papyrus *n* = 55	*p*-Value
Ellis type				
1	33 (31.1)	16 (31.4)	17 (30.9)	0.96
2	26 (24.5)	10 (19.6)	16 (29.1)	0.26
3	47 (44.3)	25 (49)	22 (40)	0.35
Dissection	27 (25.5)	10 (19.6)	17 (30.9)	0.18
No-reflow	8 (7.6)	4 (7.8)	4 (7.3)	1
TIMI 3 after PCI	89 (84)	45 (88.2)	44 (80)	0.25
TIMI 0 after PCI	5 (4.7)	2 (3.9)	3 (5.4)	1
Glycoprotein IIb/IIIa inhibitor	9 (8.6)	4 (8)	5 (9.1)	1
IABP/LVAD	5 (4.8)	2 (3.9)	3 (5.7)	1
Protamine sulphate administration	18 (17)	1 (2)	17 (30.9)	<0.001
Prolonged balloon dilatation	37 (34.9)	16 (31.4)	21 (38.2)	0.46
Transcatheter fat embolization	1 (0.9)	0 (0)	1 (1.8)	1
Non-CS implantation to seal rupture	98 (92.5)	48 (94.1)	50 (90.9)	0.72
Tamponade–echo	44 (41.5)	27 (52.9)	17 (30.9)	0.02
Pericardiocentesis	43 (40.6)	25 (49)	18 (32.7)	0.09
Emergency cardiac surgery	12 (11.3)	8 (15.7)	4 (7.3)	0.17
Cardiogenic shock	28 (26.4)	14 (27.5)	14 (25.5)	0.81
Periprocedural death	8 (7.6)	3 (5.9)	5 (9.1)	0.72
Urgency blood transfusion	19 (17.9)	9 (17.7)	10 (18.2)	0.94
Periprocedural cardiac arrest	18 (17)	9 (17.7)	9 (16.4)	0.86
Length of DAPT (months)				
- 0	2 (2.3)	1 (2.1)	1 (2.4)	0.006
- 1	4 (4.5)	1 (2.1)	3 (7.1)	
- 6	9 (10.1)	0 (0)	9 (21.4)	
- 12	74 (83.2)	45 (95.7)	29 (69.1)	

Data are presented as mean ± standard deviation or count (percentage). CS, covered stent; DAPT, dual antiplatelet therapy; IABP, intra-aortic balloon counterpulsation; LVAD, left ventricle assist device; PCI, percutaneous coronary intervention; TIMI, Thrombolysis In Myocardial Infarction.

**Table 4:** Follow-up data according to the stent graft type within 30 and 365 days.

Selected Indices	Total *n* = 106	30 Days			365 Days		
		GraftMaster *n* = 51	Papyrus *n* = 55	*p*-Value	GraftMaster *n* = 51	Papyrus *n* = 55	*p*-Value
Re-PCI	22 (20.7)	7 (13.70	3 (5.4)	0.19	11 (21.6)	11 (20)	0.84
TVR	18 (17)	9 (17.6)	2 (3.6)	0.02	12 (23.5)	6 (10.9)	0.08
TLR	16 (15.1)	9 (17.6)	2 (3.6)	0.02	11 (21.6)	5 (9.1)	0.07
Cardiac death	15 (14.1)	4 (7.8)	10 (18.2)	0.11	4 (7.8)	11 (20)	0.07
Myocardial infarction	9 (8.5)	4 (7.8)	1 (1.8)	0.19	7 (13.7)	2 (3.6)	0.08
MACE	33 (31.1)	13 (25.5)	11 (20)	0.5	17 (33.3)	16 (29.1)	0.63
Stent Thrombosis							
Acute	4 (3.8)	3 (5.9)	1 (1.8)	0.34	-	-	-
Subacute	1 (0.9)	1 (2)	0 (0)	0.48	-	-	-
Late	1 (0.9)	-	-	-	1 (2)	0 (0)	0.48
Overall	6 (5.7)	-	-	-	5 (9.8)	1 (1.8)	0.08

Data are presented as count (percentage). MACE, major adverse cardiac events; PCI, percutaneous coronary intervention; TLR, target lesion revascularisation, TVR, target vessel revascularisation.

## Data Availability

Possible disclosure of data is available on personal requests.
